# Does mating experience of male house crickets affect their behavior to subsequent females and female choice?

**DOI:** 10.1007/s00265-012-1418-0

**Published:** 2012-09-23

**Authors:** Paweł Ręk

**Affiliations:** Department of Behavioural Ecology, Institute of Environmental Biology, Faculty of Biology, Adam Mickiewicz University, Umultowska 89, 61-614 Poznań, Poland

**Keywords:** Mating experience, Female choice, Resource value, Courtship song, *Acheta domesticus*

## Abstract

Male mating experience was shown to play an important role in settling conflicts between males; however, little is known about whether and how prior access to females influences male behavior during intersexual interactions and female choice itself. Here, I experimentally test this relationship in the house cricket (*Acheta domesticus*) by combining one-on-one interaction between the male and female with direct comparison of males by the female, but precluding aggression between males. I found that solitary males were more active during subsequent courtship displays than paired males, suggesting the detrimental effect of mating on courtship performance. At the same time, females spent significantly more time close to solitary males or playbacks of male's natural courtship songs, and responded positively to the condition of males, ignoring body size of males. In contrast, females responded similarly to computer-modified playbacks of courtship songs of solitary and paired males with standardized rate of phrases and amplitudes; however, when females were additionally allowed to contact with anesthetized males they spent more time close to bigger males, irrespective of the acoustic parameters of courtship songs. These results show that although females were able to differentiate between many behavioral and morphological characteristics of males, including voluntary and intrinsic ones, they preferred traits conditional upon the costliness of male's displays. In addition, mating experience appeared to be a crucial factor in the choice of a particular costly mating strategy by males.

## Introduction

The idea that decision-making is mediated through motivation has long history in behavioral research (Lorenz 1950; Bindra 1978; Hogan 1997; Anselme 2007). Motivation is generated by the objective value of the resource (Maynard Smith 1982; Enquist 1985; Barlow et al. 1986; Enquist and Leimar 1987; Riechert 1998; Hurd 2006; Baker and Maner 2008; Bergman et al. 2010) as well as by the level of deficiency (Nosil 2002; Chancellor and Isbell 2008), i.e., the positive or negative experience with the factor. It is widely accepted that motivation makes a connection between individual's needs and the actions that are aimed at fulfilling them (Elwood et al. 1998; Grouzet et al. 2004; Anselme 2007). Also, asymmetry in motivation, except for resource holding potential (RHP) and intrinsic aggressiveness, has been one of the main parameters of models of animal communication (Barlow et al. 1986; Maynard Smith and Harper 2003; Hurd 2006). In sexual context, motivation was shown to play an important role in settling aggressive conflicts between males (Brown et al. 2006, 2007; Dissanayake et al. 2009). For example, it was shown that males having no prior access to females are more aggressive during conflicts with rivals than sexually more experienced males (Brown et al. 2006). Little is known, however, about whether and how motivation caused by differential mating experience influences male behavior during intersexual interactions and female choice itself.

Because restricted access to females intensifies male's aggressiveness during fights, the decision of the male to court female and the choice of a particular mating strategy may be based on male's motivational state (Rantala and Kortet 2003; Thomas and Simmons 2008; Zuk et al. 2008). Consequently, unmated males should be prone to invest more in courtship (Molina and Christenson 2008), for the same reason why unmated males are more aggressive during fights with rivals. Nevertheless, research to date has shown neither evidence for female choice nor differences between males' courtship behaviors based on asymmetry in their previous access to females (Brown et al. 2007). Contrary to this, it was suggested that motivational asymmetry leads to differential mating success mediated only by direct male–male aggression (Brown et al. 2007).

In this paper, I examine the potential relationship between male's previous access to females and female mate choice in the house cricket (*Acheta domesticus*). I hypothesize that the courtship behavior of males is a costly handicap, providing females with information about male's quality. Such a system might evolve through indirect selection both because of a correlation among the costliness of courtship behavior, male viability, and female preferences (Andersson 1994) and as result of Fisherian runaway selection; however, courtship song might just as easily indicate some direct material benefit to females (e.g., male territory quality). The finding that courtship behavior directed at females and aggression towards males are both based on the same male's motivational state might give rise to the notion that in crickets, depending on local circumstances and RHP of a male, courtship and aggression represent two alternative reproductive costly strategies.

**Fig. 1:**
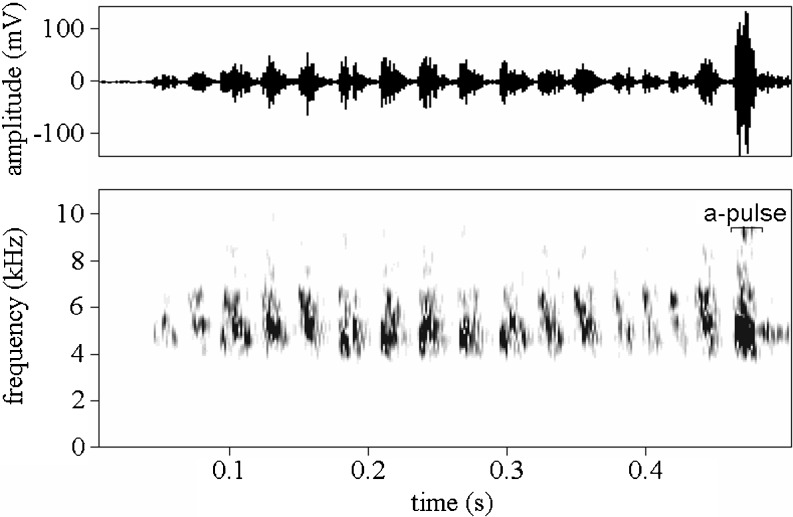
A phrase of the courtship song of *Acheta domesticus* consisting of numerous low frequency pulses and a single high frequency a-pulse

In crickets, male's courtship is a complex behavior, which in addition to song, comprise visual, tactile, and chemical cues and signals (Hack 1998). Calling song is used to attract females at a distance, and it is known to play an important role in female mate choice (Stout et al. 1983; Gray 1997; Wagner and Reiser 2000). By contrast, courtship song is produced only during interactions between males and females, once they are in direct contact. It has specific power spectrum and oscillogram (Fig. 1) (Nelson and Nolen 1997) and is costly to produce (Hack 1998). It was shown in the field cricket (*Teleogryllus oceanicus*) that courtship song is very variable between males and suggested that its role is giving information about individual characteristics (Zuk et al. 2008); however, the nature of those differences has not been revealed. The courtship song has recently been recognized as being important for female's mate choice in crickets (Killian and Allen 2008; Zuk et al. 2008), and in many species, courtship song is necessary to elicit normal levels of female mounting of the male during courtship (Nelson and Nolen 1997; Rantala and Kortet 2003; Killian et al. 2006; Thomas and Simmons 2008; but see Zuk et al. 2006).

To understand the relationship between male's courtship behavior and female mate choice, it is necessary to conduct an experiment combining one-on-one interaction between a male and female with direct comparison of males by females but precluding physical aggression between males. It is because intrasexual aggression can effectively inhibit intersexual selection (Loher and Dambach 1989; Wong and Candolin 2005). Nevertheless, thus far in none of the studies, the effects of direct comparison of males by females were tested controlling for the effects of male–male aggression. I carried out the experiment on female crickets, assigned to five stimuli groups with the asymmetry in male's previous access to females as the main experimental factor. In this experiment, females had simultaneous access to two males, while males were physically isolated from one another. The main goal of such an experiment was to associate female behavior with a suite of potential male stimuli present during courtship, considering the voluntary (i.e., motivation based) and intrinsic (i.e., RHP based) character of preferred stimuli. The distinction between voluntary and intrinsic mechanisms of signaling can have strong implications for the reliability of signaling systems. Some signals, for example, can be honest intrinsic correlates of physical and genetic attributes of senders (so called indices) in which a signal's meaning is associated with its method of production. Others can function as costly performance displays, where the link between the signal and male's genetic quality is indirect and which enable some level of dishonesty resulting from voluntary adaptive choices (Maynard Smith and Harper 2003). Knowledge of constraints on signal production during male–female interactions is therefore crucial for our understanding of both sexual selection and the evolution of animal communication.

In the first stimulus group, females were given access to courting males, which aimed at the assessment of whether mated and unmated males differ in their behavior to subsequent females and their ability to attract a female. Although the study design prevented males from coming into physical contact, males were very likely in acoustic contact. For this reason, there is no way to say that the results of such a stimulation are exclusively due to female choice or male–male acoustic competition. To distinguish between these alternatives, in the second group, females listened to playbacks of males' natural courtship songs, recorded during isolated male–female encounters. Because females can simultaneously attend signals in other modalities (i.e., pheromones) in addition to acoustic ones (Tregenza and Wedell 1997; Kortet and Hedrick 2005; Thomas and Simmons 2008; Rebar et al. 2009) once they have made contact with a courting male, this stimulation, with reference to the first one, aimed at controlling the influence of acoustic parameters of males' behavior on female choice. Previous studies of female preferences for courtship song (Balakrishnan and Pollack 1997; Wagner and Reiser 2000; Rantala and Kortet 2003; Tregenza et al. 2006) predicted that females would prefer songs with a higher duty cycle. To control for the effect of duty cycle, females from the third group listened to playbacks of computer modified songs of males, with equalized duty cycles (similar duration of sound per unit time) and amplitudes. Females from the fourth and fifth groups also listened to playbacks of computer modified songs, but additionally, they were allowed tactile contact with anesthetized males. In addition, in the fourth group, the songs were played back correctly (came from given anesthetized males), whereas in the fifth group, the songs were played back crosswise with anesthetized males, so that acoustic and tactile/visual information about males must have been contradictory for females in the fifth group. The function of the last two stimuli groups was to control the marginal influence of male's RHP predictors and pheromone profiles, available through chemical or tactile cues, on female choice. If courtship experience of males is communicated, I expect females to express nonrandom choice with regard to male's previous access to females.

## Methods

### Cricket culturing

The crickets were from a laboratory stock and were maintained at 22–28 °C on a 13:11 h light:dark cycle that was set for lights off at 8 pm. Initially, the crickets were kept in 2,500-l containers with a wet base (coconut fiber) and carton shelters. Food and water was provided ad libitum and consisted of dry cat food (30 % of meat), porridge, and leaves of dandelion. Males and females were isolated as juveniles before they matured. Only females receptive to courtship songs and males able to produce courtship songs were selected for the study.

### Experimental conditions and stimuli

The research were carried out between 17 and 23 September 2008 and consisted of 125 independent trials. Trials were carried out in a plastic container, empty from above, and consisting of three chambers: central one (30 l/10 w/10 h [cm]), where the female was placed during the examination and two side chambers separated with wire screens (10 l/10 w/10 h each), where males were placed and/or from where the recorded samples were played back to females. Wire screens were practically transparent (dot 2/2 mm, thickness of the wire 0.1 mm) and did not prevent antennal contact between a male and female. As a result, females could choose between two simultaneously courting males or playbacks but there was no physical aggression allowed between competing males. Between tests, the arena was wiped with a sponge moistened with 70 % ethyl alcohol to eliminate odor cues left by previous males and females. Trials took place in the darkened room with a stable temperature of 26 °C. The cage was lit with an overhead red 15 W bulb.

Males used for testing the reactions of females (*n* = 250) were divided into two groups (treatment) with experimentally manipulated access to females prior to testing: paired males and solitary males (*n* = 125 each). During manipulation, males were individually housed in glass containers (15 l/12 w/12 h [cm]) with food and an egg carton shelter. Paired males received a receptive female for one-night encounters. Solitary males also received a receptive female, but she was separated with a wire screen, as a control for energy expenditures by males during courtship. Females were let in at 5 pm and removed the next morning at 7 am. The receptivity of such females was determined during a pre-test with different males, 1–2 h before the females were used. Only females that tried to mount a male were used for manipulation of males. During each trial (*n* = 125), different paired and solitary males were used. Furthermore, males from both groups were randomly but proportionally assigned to three categories: males actively courting females during trials (*n* = 50), males recorded and anesthetized (*n* = 100), and males recorded (*n* = 100).

Experimental females (*n* = 125) were randomly assigned to five stimuli groups (25 females each). Group 1 (S1)—with two males, one paired and one solitary, allowed to curt the female from behind the wire screens. Group 2 (S2)—with two natural playbacks, one recorded from a paired male and one recorded from a solitary male, played from behind the wire screens. Group 3 (S3)—with two computer modified playbacks, one recorded from a paired male and one recorded from a solitary male, played from behind the wire screens. Group 4 (S4)—with two computer modified playbacks, one recorded from a paired male and one recorded from a solitary male, played from behind the wire screens, and with two males anesthetized on ice for 10 min, placed close to wire screens back to the female with their wings raised and held in place by wax (a compound of beeswax and resin, spread between and at the base of tegmina), which is a natural posture of males during courtship song production. Playback calls came from the given anesthetized males. Group 5 (S5)—with two computer modified playbacks, one recorded from a paired male and one recorded from a solitary male, played from behind the wire screens, and with two males anesthetized on ice for 10 min placed close to wire screens back to the female with their wings crested. Playback calls came from the anesthetized males, but the paired playback was played from the side of the solitary male and vice versa. During an experimental session, trials with different stimuli were carried out in random order.

Males from S1, S4, and S5 were manipulated during the night before the experimental sessions (5 pm–8 am). Assignment of males to S1, S4, and S5 trials took place about half an hour before the trials. During that time, S4 and S5 males were recorded and anesthetized. Males from S2 and S3 were manipulated and recorded a few days before the samples were used because these males did not participate directly in trials; however, the recording of samples was randomly distributed during the day after manipulation of males to provide similar distribution of the time between manipulation and recording (S2–5) or active participation of males (S1) in all groups of males. The age of the males while recorded was similar in all groups (6–9 days). Females were 6–11 days old during trials.

### Playback preparation

Recordings were made in the same containers as males had been manipulated. Before recording, each male received a new receptive female separated with a wire screen. Songs were recorded for a maximum duration of 6 min per male using an Sennheiser K6/ME67 microphone and a Marantz PMD670 solid-state recorder and digitized using an Avisoft SASLab Pro 4.5 (Specht 2007) sound analysis package (48 kHz/16 bit PCM files). Two types of playback were broadcast during the experiment: natural and computer-modified ones. Natural playbacks contained 5 min of an uninterrupted section of a recording of male's courtship song, starting with the beginning of song in the whole recording. Computer-modified playbacks were created by (1) removal of pauses longer than 1 s from a recording and (2) cutting a 5-min section or replication if the recording was shorter than 5 min. Consequently, although computer-modified and natural playbacks contained courtship songs with natural phrases (see Fig. 1 for definition), i.e., phrases with natural pulse rate, computer-modified playbacks were characterized by a higher phrase rate.

### Experimental protocol and data collection

The trials lasted 5 min. Before the trial, a randomly chosen female was placed in the center of the central chamber and covered with the plastic cup for 3 min for acclimatization. Males from S1 were placed in side chambers soon after the female and were let to walk freely by the beginning of and throughout the trial. Anesthetized males from S4 and S5 were placed about 5 mm from the wire screen separating females, back to the screen, and with wings crested. Such manipulations were to imitate the natural posture of males during courtship. The trials started after the female was released. In S2–5, the release of the female was simultaneous with the beginning of the playback. For playbacks, two high-frequency loud speakers (2,000–20,000 Hz), connected to the laptop computer, were placed at the end of the side chambers of the experimental container. I calibrated the sound pressure level of playbacks to 90 dB at a distance of 10 cm from the speaker using a CHY 650 (Ningbo, China) sound pressure level meter. The central chamber of the experimental arena was visibly (contrasting lines) divided into three sections: central section (10 × 10 cm), where the female was initially placed and two identical side sections (10 × 10 cm each). The preference for paired and solitary stimuli was defined by the time spend by the female in a side section adjacent to a given stimulus; however, it was the crossing of the border between sections by the female's head that switched the timer on/off.

Except for the behavior of females, I controlled for the behavior and acoustic parameters of courtship songs of males. During the S1 trials, I collected the data on male activity: latency to first stridulation, percent of time spent on stridulating, and percent of time spent on walking. In playbacks broadcast during S2, I noted the percent of time with stridulation by males. Additionally, in all recordings, I calculated the average number (number per minute) of high-energy pulses, so called a-type pulses (Fig. 1) (Nelson and Nolen 1997) in courtship songs. Because a-pulses are energetically expensive, this parameter is likely to be condition dependent and be subject to female preference. The body mass (with electronic balance, ±0.001 g) and the femur length (with digital caliper, ±0.01 mm) of the males from all treatment groups were measured after recordings. In the analyses, femur length of males was used as an estimate of body size and residual of regression of body mass on femur length was used as an estimate of condition of males.

### Statistics

I used generalized estimating equations (GEE) to analyses the influence of the male treatment (paired vs. solitary), stimuli (S1–5), male body size, and male condition on the time spent by females in different sections of the experimental area. Within the model, the within-subject correlation between the time spent by females close to either of the two stimuli was controlled, with female used as the subject variable (*n* = 125) and treatment (paired vs. solitary) used as the within-subject variable. In the analysis, only the main factors and first degree interactions were used. Because evaluation of main effects is not permissible when there are significant interactions (Engqvist 2005), I also performed a separate analysis for each stimulus group to test the response of the dependent variable to the covariates (male body mass and male condition). All of the analyses were conducted in SPSS 19. All *p* values are two-tailed. If not stated otherwise, means ± SE are given.

## Results

### Description of males and stimuli

Neither femur length nor condition differed significantly between solitary and paired males (dependent *t* test; femur length: *t* = 0.38, df = 124, *p* = 0.71; condition: *t* = 1.39, df = 124, *p* = 0.17) and between males used in different stimuli groups (ANOVA; femur length: *F*_4,245_ = 0.88, *p* = 0.48; condition: *F*_4,245_ = 0.84, *p* = 0.50). Moreover, solitary and paired males had similar incidence of a-type pulses in courtship songs (solitary: 13.52 ± 2.21 n/min; paired: 15.56 ± 2.55 n/min; *n*_solitary_ = 125, *n*_paired_ = 125; Mann–Whitney *U* test: *U* = 7557.5, *p* = 0.66).

During S1, songs of solitary males had a higher duty cycle (seconds of courtship song/trial) than songs of paired males (solitary: 114.88 ± 16.42 s; paired: 42.08 ± 10.58 s; Wilcoxon matched pairs test: *Z* = 2.57, *p* = 0.01), solitary males spent more time on walking (solitary: 145.6 ± 16.39 s; paired: 65.44 ± 12.12 s; Wilcoxon matched pairs test: *Z* = 2.91, *p* = 0.004), and had shorter latencies to courtship song than paired males (solitary: 89.08 ± 17.10 s; paired: 160.88 ± 21.55 s; Wilcoxon matched pairs test: *Z* = 2.28, *p* = 0.023). During S2, natural playbacks from solitary males had a higher duty cycle than natural playbacks from paired males (solitary: 157.4 ± 17.89 s; paired: 108.44 ± 10.23 s; Mann–Whitney *U* test: *U* = 204, *p* = 0.035).

### Experiment

**Fig. 2:**
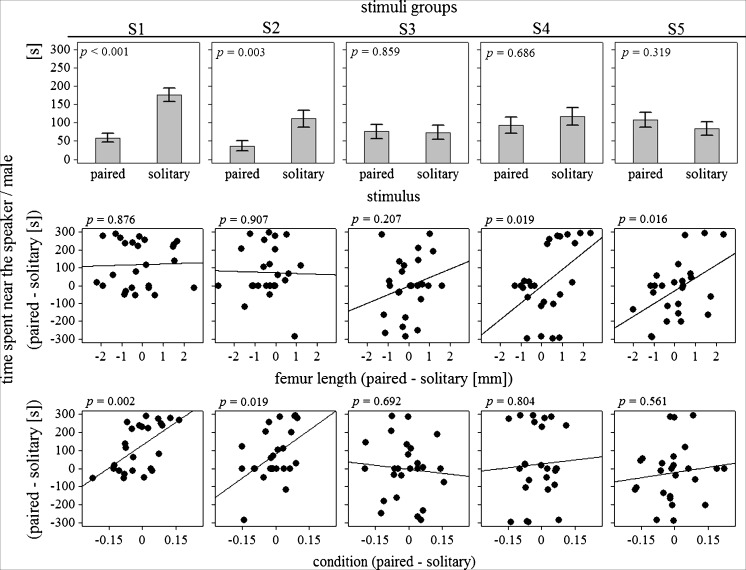
Preferences of females with reference to the treatment (paired males vs. solitary males), femur length, and condition of males in five stimuli groups. *Boxplots* show real times spent with the two males on the *y* axis. Scatterplots show the difference times spent with the two males on the *y* axis versus the difference in either femur length or condition on the *x* axes of these plots. Differences were calculated in a consistent manner for all plots, i.e., with paired minus solitary males, so that positive values mean more time with the paired male (*y* axis) and that paired male is bigger/in better condition (*x* axis)

In order to test the influence of male's previous access to females on reproductive decisions of females, I compared reactions of females to variable components of the behavior of mated and unmated males (Table 1). The key outcome of the model is the stimuli × treatment interaction term (Table 1), which explains the significant difference between responses of females to paired and solitary males and playbacks in S1 and S2 but not in the remaining groups (Table 2; Fig. 2). Generally, in all stimuli groups, females spent more time close to solitary males/playbacks than to paired males/playbacks (solitary: 112.58 ± 9.63 s; paired: 74.33 ± 8.23 s); however, this difference was due to strong preference of females to solitary males (S1) and playbacks of solitary male courtship song (S2) (Table 2; see Fig. 2 for time values in each stimuli group separately). In S3–5, females responded similarly to both types of stimuli (Table 2; Fig. 2). The second outcome of the model is the stimuli × condition interaction term (Table 1), which explains the preference of females to the condition of males in S1 and S2 but not in S3–5 groups (Tables 2 and 3; Fig. 2). In S1 and S2, the time spent close to a given male or playback was positively correlated with the male's condition, whereas in the remaining stimuli groups, this relationship was insignificant (Table 2; Fig. 2).

**Table 1:** Factors associated with preferences of females

	Wald *χ* ^2^	df	*p*
Intercept	6.31	1	0.012
Stimuli	8.20	4	0.085
Treatment	0.40	1	0.525
Femur	12.24	1	<0.001
Condition	1.64	1	0.200
Stimuli × treatment	27.34	4	<0.001
Stimuli × femur	7.91	4	0.095
Stimuli × condition	16.25	4	0.003
Treatment × femur	0.17	1	0.677
Treatment × condition	0.15	1	0.695
Femur × condition	1.13	1	0.287

GEE model including treatment (paired vs. solitary males), stimuli (S1–5), femur length of males, and condition of males. Dependent variable: time spent near the speaker/male

**Table 2:** Factors associated with preferences of females for each stimulus category separately

	S1		S2		S3		S4		S5	
	Wald *χ* ^2^	*p*	Wald *χ* ^2^	*p*	Wald *χ* ^2^	*p*	Wald *χ* ^2^	*p*	Wald *χ* ^2^	*p*
Intercept	0.01	0.909	0.10	0.746	1.69	0.194	9.73	0.002	5.30	0.021
Treatment	36.40	<0.001	8.69	0.003	0.01	0.929	0.11	0.742	0.87	0.352
Femur	0.93	0.336	0.001	0.973	2.41	0.121	14.06	<0.001	7.91	0.005
Condition	33.24	<0.001	8.34	0.004	0.01	0.916	0.31	0.577	0.45	0.504

GEE models including treatment (paired vs. solitary males), femur length of males, and condition of males. Dependent variable: time spent near the speaker/male. All df = 1. Interaction terms were insignificant and removed prior to evaluating the main effects

**Table 3:** Preferences of females towards condition and body size of males in each stimulus category separately

Stimulus (*n*)	Preference	Femur (mean ± SE)	Condition (mean ± SE)
S1 (23)	+	11.54 ± 0.13	$$ \left. {\matrix{ {0.0{1}0\pm 0.0{14}} \hfill \\ { - 0.0{2}0\pm 0.0{1}0} \hfill \\ } } \right\} $$
	−	11.72 ± 0.18	
S2 (16)	+	11.49 ± 0.16	$$ \left. {\matrix{ {0.0{18}\pm 0.0{14}} \hfill \\ { - 0.00{6}\pm 0.0{1}0} \hfill \\ } } \right\} $$
	−	11.49 ± 0.11	
S3 (19)	+	11.56 ± 0.14	0.007 ± 0.013
	−	11.38 ± 0.10	0.009 ± 0.013
S4 (21)	+	$$ \left. {\matrix{ {{11}.{71}\pm 0.{14}} \hfill \\ {{11}.{3}0\pm 0.{17}} \hfill \\ } } \right\} $$	−0.001 ± 0.013
	−		0.006 ± 0.013
S5 (21)	+	$$ \left. {\matrix{ {{11}.{57}\pm 0.{16}} \hfill \\ {{11}.{23}\pm 0.{17}} \hfill \\ } } \right\} $$	0.015 ± 0.014
	−		0.010 ± 0.013

“+” and “−” refer to the time (longer and shorter respectively) spent by a female close to the given stimulus during a trial, provided that there was a difference; “}”—significant differences

The presence of motionless males in S4 and S5 enabled females to assess the size of males, but it prevented females from estimating the condition of males. Consequently, male condition did not influence decisions of females in S4 and S5, whereas the size of males did (Tables 2 and 3; Fig. 2). It should be noted, however, that despite the general significance of male size effect (Table 1), only S4 and S5 females revealed significant preferences for bigger males (Tables 2 and 3; Fig. 2). Furthermore, none of the factors considered affected significantly the behavior of S3 females (Tables 2 and 3; Fig. 2).

## Discussion

I investigated the role of male's previous access to females in female mate choice. Firstly, the experiment showed that solitary (prevented from mating) males were more active during courtship than paired (allowed access to receptive females) males. Secondly, the results indicated that such a variability in male's behavior affected female choice. Females spent significantly more time close to solitary than paired stimuli; however, such an inclination was conditional upon whether a stimulus contained cues of the costliness of the display (only S1 and S2). The most energetically costly mating behaviors of male crickets are walking and courtship calling in order (Hack 1998), and solitary and paired stimuli differed either in the time spent on walking and duty cycle of courtship song (S1) or the duty cycle of courtship song only (S2). Therefore, the fact that females spent more time close to solitary stimuli in S1 than in S2 (Table 2) and that the choice of females was positively influenced by the condition of males in S1 and S2 (Table 1; Fig. 2) strongly support the prediction that courtship behavior of male crickets provides females with important information about the costliness of male's display. This result is in contrast with the earlier data from field crickets (*Gryllus texensis*), indicating that courtship song does not reflect male condition despite its energetic cost (Gray and Eckhardt 2001). Because of higher duty cycles of solitary S2 playbacks, the difference between the present and earlier study could not result solely from the effect of competition between simultaneously courting males in S1.

In groups, where the difference between solitary and paired playbacks was limited to the acoustic structure of courtship songs (S3) or the acoustic structure of courtship songs and male visual (size) and chemical parameters (S4 and S5), the effect of previous access to females was undetectable, which excludes the potential relationship between the morphological or chemical parameters of males (e.g., pheromone profiles) and their mating history. In contrast, in S4 and S5, females preferred bigger males despite apparently ignoring the size of males in S1–3. This result suggests a specific hierarchy of males' characteristics for female choice. With the lack of variability in preferred characteristics, females concentrated on secondary parameters but still sufficient ones to make a choice on. At first sight, the choice of male condition before male size may appear surprising (Savage et al. 2005; but see Shackleton et al. 2005), but since crickets frequently communicate in the darkness, their acoustic endurance may have higher positive impact on the fitness than their body size.

The results did not reveal female choice based on specific signals, i.e., behaviors that evolved specifically for their signal value, not as byproducts of selection for some other function, which may imply the low cost of choosiness for females. For example, solitary and paired males did not differ with respect to the frequency of a-pulses, despite a song with more such pulses requires more energy (Hoback and Wagner 1997; Hack 1998). Conversely, decisions of females appeared to rely on a broad spectrum of simple cues predictive of male condition, such as the total time spent on stridulating (duty cycle) or body mass in case of the lack of differences in duty cycle. Such multiple cues may be assessed in an additive way and serve to reduce the cost of mate choice by making the evaluation of a prospective mate easier (Candolin 2003). However, it should not be excluded that the structure of the courtship songs contained some signals important for females. In S3, where females neither had direct access to males nor to energetically diversified playbacks, females revealed some inclination toward bigger males (Fig. 2). Although this effect was insignificant, it may suggest that the structure of courtship song contains some unknown parameters associated with the size of males, the same as in the advertisement (calling) song (Gray 1997; Ryder and Siva-Jothy 2000).

The higher investments of unmated males in courtship imply that an already-mated male puts less value on the next female he encounters than the male who hasn't mated.

By contrast, because male crickets can regenerate a spermatophore within at least 1 h, all males were undoubtedly capable of inseminating the female. Therefore, it seems that the treatment controlling for the access to females manipulated subjective value of females for males rather than affected reproductive function of males. Resource availability is one of the primary factors affecting animals' subjective resource value (Enquist and Leimar 1987). Asymmetry in resource value is in turn one of the best predictors of the behavior of males and females during intrasexual interactions (Humphries et al. 2006; Hurd 2006; Tibbetts 2008; Dissanayake et al. 2009; Bergman et al. 2010). (Judge et al. 2010) revealed in *Gryllus pennsylvanicus* that mating itself affects negatively male success in aggressive contests, which together with these results suggests that, irrespective of the accepted reproductive strategy or circumstances, mating is detrimental for male's subjective resource value.

Although the results suggest the sensitivity of females to costly displays of males, it is not clear whether such an association functions in natural conditions. In fact, previous laboratory research has shown that males' mating experience is expressed only during aggressive contests (Brown et al. 2007), whereas for females to benefit from mate choice, there must be detectable differences among males in the benefits they offer or the costs they impose (Pitnick and García-González 2002). However, this study is not intended to estimate the importance of benefits females may collect from being sensitive to male courtship, it is widely accepted that females with increased efficiency of choice enjoy strongly elevated fitness compared to females with reduced choice efficiency (Maklakov and Arnqvist 2009). Hence, potential benefits and low costs may explain the observed behavior of females; even if it is rare in natural conditions. It is reasonable to assume that in natural conditions, most of males are completely inexperienced (virgin) only for a short period of time, and that a majority of male–female encounters involve experienced males; however, any two males are unlikely to be equally experienced and motivated in the wild, and such differences may potentially play a significant role for sexual selection and mating success of females in the wild.
